# Crash data integration for road safety analysis in Iran

**Published:** 2019-08

**Authors:** Ali Afshar

**Affiliations:** ^*a*^Cambridge Systematics, Inc.

## Abstract

Roadway crashes have put a huge toll on the country’s resources in the last three decades. Since early 2000, roadway crash fatalities in Iran became alarming for international observers and eventually in 2005, WHO came to rescue by providing expertise and resources to the Iranian governmental bodies and authorities to draw a road-map and to tackle this national issue. The government has passed appropriate legislation to facilitate the process for authorities to plan, implement, manage, and enforce what the road-map envisioned. Meanwhile Universities, NGOs, and media put more resources to dissect the problems, develop consensus among stakeholder and to identify areas that need public awareness and participation for moving toward safer transportation system. Present study looks at the samples of actions taken by authorities to scan the progress, lessons learned and to identify steps for improvements in accuracy and efficiencies. It is found that all stakeholders involved in roadway crashes (Driver, Vehicle, and Road) are recording appropriate attributes but because these data are not connected, detailed analysis and meaningful evaluations are lacking reliability and trust worthiness. Technological advances on data fusion, big data analysis, and relational databases allow bringing all parties resources/databases into a readable platform which requires minimal effort compare to the benefits it offers to all stakeholders. Data queries from this platform would enable analysts to produce meaningful reports for decision maker to dedicate adequate resources for combating this national tragedy and assisting stakeholder to plan toward zero deaths.

**Keywords:**

Crash Data, Integration, Road Safety Analysis

